# Fabrication of pH-Sensitive Tetramycin Releasing Gel and Its Antibacterial Bioactivity against *Ralstonia solanacearum*

**DOI:** 10.3390/molecules24193606

**Published:** 2019-10-07

**Authors:** Xiaozhou Ma, Shunyu Xiang, Huijun Xie, Linhai He, Xianchao Sun, Yongqiang Zhang, Jin Huang

**Affiliations:** 1 School of Chemistry and Chemical Engineering, Southwest University, Chongqing 400715, China; maxiaozhou@swu.edu.cn (X.M.); h996130557@163.com (L.H.); 2 Chongqing Key Laboratory of Soft-Matter Material Chemistry and Function Manufacturing, Southwest University, Chongqing 400715 China; xiangshunyu0325@163.com (S.X.); sunxianchao@163.com (X.S.); 3 College of Plant Protection, State Cultivation Base of Crop Stress Biology for Southern Mountainous Land, Southwest University, Chongqing 400715 China; xiehj941019@163.com

**Keywords:** pH-dependent pesticide releasing, double-crosslinking gel, alginate gel, tetramycin releasing, *Ralstonia solanacearum*

## Abstract

*Ralstonia solanacearum* (*R. solanacearum*)-induced bacterial wilt of the nightshade family causes a great loss in agricultural production annually. Although there has been some efficient pesticides against *R. solanacearum*, inaccurate pesticide releasing according to the onset time of bacterial wilt during the use of pesticides still hinders the disease management efficiency. Herein, on the basis of the soil pH change during *R. solanacearum* growth, and pH sensitivity of the Schiff base structure, a pH-sensitive oxidized alginate-based double-crosslinked gel was fabricated as a pesticide carrier. The gel was prepared by crosslinking oxidized sodium alginate (OSA) via adipic dihydrazide (ADH) and Ca^2+^. After loading tetramycin into the gel, it showed a pH-dependent pesticide releasing behavior and anti-bacterial activity against *R. solanacearum*. Further study also showed that the inhibition rate of the tetramycin-loaded gel was higher than that of industrial pesticide difenoconazole. This work aimed to reduce the difficulty of pesticide administration in the high incidence period of bacterial wilt and we believe it has a great application potential in nightshade production.

## 1. Introduction

Bacterial wilt is a typical soil-borne plant disease, which has a wide geographical distribution and causes great losses in agricultural production of the nightshade family annually [1,2,3,4]. As a kind of pathogen of bacterial wilt, *Ralstonia solanacearum* (*R. solanacearum*) is usually hard to manage because of its infection process towards plants. Briefly, *R. solanacearum* infects plant roots through wounds or natural fissures, colonizes the plant vasculature, and produces a large number of extracellular polysaccharides (EPS), leading to wilting and death of the host plant [5]. After the host plant is destroyed, the bacterium returns to the soil through saprophytism until it contacts new hosts [6,7]. This infection process makes it difficult to release pesticide accurately, as it will be always too late to release pesticides when symptoms of plants have been observed, but will also waste pesticide if the pesticide is released too early before the growth of the *R. solanacearum* [8,9,10]. Furthermore, the overlapping of the bacterial wilt period and rainy season make the management of the disease much harder because of the pesticide wastage induced by the rain, and usually requires a great number of pesticides to prevent the disease annually, which may cause serious environmental pollution [11]. On the other hand, despite the soil pH decrease induced by other factors (such as industrial production or improper fertilizer usage), it has been found that the growth of *R. solanacearum* will always cause soil acidification, which is due to the acidic components produced by the bacteria [12]. Meanwhile, studies also have indicated that the environmental factor-induced soil acidification will also increase both the growth and infection ability of *R. solanacearum* [13]. Specifically, it has been reported that the proportion of infected soils with a pH lower than 5.5 was much higher than that of non-infected soils, and also the seriousness of the bacterial wilt was increased when the soil pH was around 5.5. Thus, an efficient way to prevent the bacterial wilt while decreasing the pesticide releasing amount may be to fabricate a pesticide carrier that can prevent the pesticide waste caused by the rain on the one hand, while on the other hand, being able to auto-adapt its pesticide releasing rate with the presence of *R. solanacearum* to prevent the bacterial wilt of nightshade plants. An appropriate indicator of this presence is soil pH.

Hydrogel has been widely applied in drug releasing [14,15,16,17]. After being packed into the hydrogel, the releasing behavior of drugs can be controlled on the basis of the high design ability and fast substance exchange of hydrogels. As environmental pH has been proven as an important indicator of some diseases of plants, pH-sensitive hydrogels for pesticide releasing have been developed. The main method of such hydrogel-based drug carriers are to crosslink the hydrogel via certain pH-sensitive physical interactions or chemical bonds; these crosslinking points can be broken up when the environmental pH changes, thus causing the release of the packaged pesticides in the carrier to manage diseases accurately [18]. For example, the chemical interaction of chlorpyrifos and polydopamine could be affected by environmental pH. Thus, by using chlorpyrifos, polydopamine, attapulgite, and alginate, an alginate gel-based nanocarrier for chlorpyrifos releasing can be fabricated. The carrier presents a high releasing rate under pH 8.5 to release chlorpyrifos [19]. However, to our knowledge, the development of a pH-sensitive pesticide carrier for bacterial wilt management is still a problem that remains to be solved.

Tetramycin is a kind of pesticide that has shown a wide anti-microorganism spectrum, including *R. solanacearum* and *Phytophthora capsici*, among others [20,21,22,23,24,25]. Herein, by adding tetramycin into oxidized sodium alginate (OSA), and crosslinking OSA with adipic dihydrazide (ADH) and calcium ions (Ca^2+^), tetramycin could be loaded into an OSA-based double crosslinking gel. On the basis of the pH sensitivity of Schiff base, the releasing rate of tetramycin could be controlled by the environmental pH, while a Ca^2+^-based crosslinking point could be used to elongate the effective pesticide duration of the tetramycin. Thus, as Schiff base between ADH and OSA can be hydrolyzed when the environmental pH is lower than 6, the gel could perform a moderate pesticide releasing rate when the environmental pH is above 6, while increasing its releasing rate when the pH decreases below 6 [26,27,28,29,30]. The ability of tetramycin-loaded ADH-OSA gel to manage *R. solanacearum* under different environmental pH controlled in a lab setting was also studied in this work. Considering that this pH-sensitive pesticide releasing behavior was coincident to the pH change during the growth of *R. solanacearum*, and the biodegradation of OSA, this gel could be applied in the management of bacterial wilt while providing a new pH-sensitive pesticide releasing platform to improve the efficiency of pesticide application in agricultural production.

## 2. Results and Discussion

### 2.1. Preparation of Tetramycin-Loaded ADH-OSA Gel

**Figure 1:**
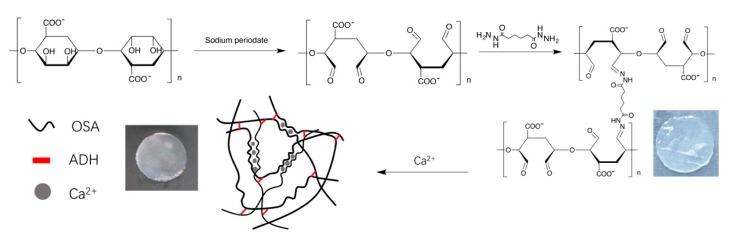
The schematic of fabricating adipic dihydrazide crosslinked oxidized sodium alginate (ADH-OSA) gel.

**Figure 2:**
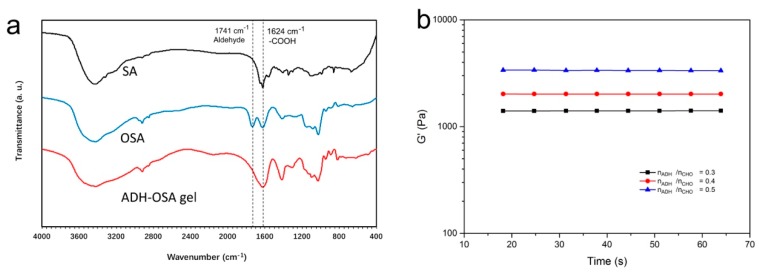
(**a**) The FTIR spectra of sodium alginate (SA), oxidized sodium alginate (OSA), and ADH-OSA gel. (**b**) The storage modulus of tetramycin-loaded gel with different linker amounts. n_ADH_ and n_CHO_ are the molar quantity of adipic dihydrazide (ADH) and total CHO group in the gel, respectively.

Our strategy is shown in Figure 1. OSA was firstly prepared according to the literature [31], and was mixed with ADH to fabricate the pre-gel. The pre-gel was then merged into the Ca^2+^ solution to make the final OSA-ADH gel. After mixing ADH and OSA at pH 7.5 for 30 min, a transparent pre-gel was formed. The pre-gel was then merged into the Ca^2+^ solution for 5 min to make the final product. Figure 1 shows the image of both pre-gel and ADH-OSA gel. The gel was characterized by FTIR, and the spectra were shown in Figure 2a. From the figure, it could be found that compared to the sodium alginate, a new peak located at 1741 cm^−1^ could be observed in the FTIR spectrum of OSA that was attributable to the vibration of aldehyde groups, indicated by the successful introduction of aldehyde functionalities [32]. Furthermore, when ADH and OSA were mixed and merged into the Ca^2+^ solution to form ADH-OSA gel, it was found that the two peaks of aldehyde groups and carboxyl groups were merged into one large band, attributable to the appearance of the peak located around 1650 cm^−1^ assigned to the stretching vibration of C=N bonds, which suggests the formation of a Schiff base bond in the gel [32,33].

The effect of the amount of ADH applied in the preparation of ADH-OSA gel upon the gel mechanical property was also studied. Figure 2b shows the storage modulus (G’) of ADH-OSA gels with different ADH amounts. With the increase of the molar ratio of ADH and aldehyde groups of OSA (n_ADH_/n_CHO_), it could be seen that the G’ of the gel also increased, which was due to the increase of the amount of crosslinking points formed in the gel. Meanwhile, the loss modulus of the gel also decreased with the increase of n_ADH_/n_CHO_ value (Figure S1) due to the limitation of the increased crosslinking point in the gel network to the degrees of freedom of OSA molecules. However, after merging pure ADH-OSA gel into double distillated H_2_O (ddH_2_O), the pH of the leachate did not change, indicating that the application of the gel would not affect the environmental pH (Figure S2).

On the other hand, as the calculation of the tetramycin concentration was crucial to the following study of the pesticide-releasing behavior and the antibacterial ability of the tetramycin-loaded gel. Thus, the standard curve of tetramycin was built to facilitate the calculation of tetramycin. During the preparation of the standard curve, tetramycin was dissolved into the ddH_2_O until the concentration reached 0, 25, 50, 200, or 400 μg/mL, and the solution was analyzed by the UV-VIS spectrometer. The result is shown in Figure S3—the ultraviolet (UV) absorbance of tetramycin at a wavelength at 272 nm was highly correlated to its concentration, showing an extra high linear relationship, and the *R*^2^ of the curve was able to reach as high as 0.9999. This indicated that the curve could be used to calculate the tetramycin concentration in the solutions.

### 2.2. Affection of Environmental pH to the Tetramycin Releasing Rate

**Figure 3:**
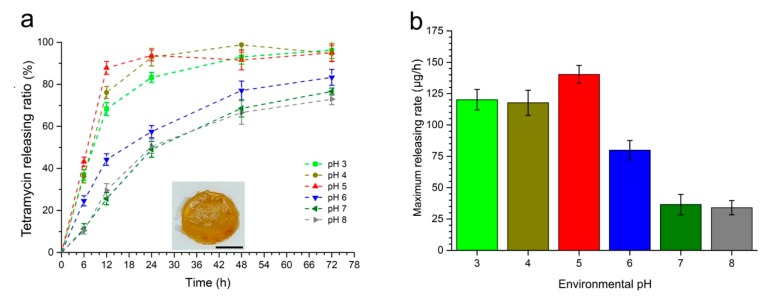
(**a**) The tetramycin-releasing ratio of the gel (n_ADH_/n_CHO_ = 0.3) under different pH conditions; the insertion was the tetramycin-loaded gel, scale bar = 0.2 cm. (**b**) The maximum releasing rate of the gel under different pH conditions.

Theoretically, according to the strategy, after being loaded into the gel, tetramycin molecules would be gradually released when the gel was immersed into the phosphate buffer (PB) at pH 7. However, when the pH of the buffer was decreased, the releasing rate of the tetramycin would be increased because of the hydrolysis of the Schiff base at the crosslinking point of the gel. Thus, tetramycin was firstly loaded into the ADH-OSA gel. According to the range of soil pH change during the bacterial wilt, the tetramycin releasing rate of ADH-OSA gel (n_ADH_/n_CHO_ = 0.3) under the pH range of 3 to 8 was analyzed (Figure 3a). From the figure, ADH-OSA gel performed the lowest releasing rate when the environmental pH was at 7 and 8, less than 80% of the loaded pesticide was released in 72 h. With the decrease of the environmental pH, the releasing rate of the tetramycin was increased. The highest releasing rate of the gel could be achieved when the environmental pH was 5. Almost 98% of the loaded tetramycin could be released in 24 h. This high-releasing rate was mainly due to the hydrolysis of the Schiff base structure in the gel. However, lower pH can make the releasing rate of the tetramycin slightly decrease, which may be induced by the change of the interaction strength between Ca^2+^ and OSA molecules. Meanwhile, from Figure 3a, it could found that the maximal releasing rate could be reached as soon as the gel was put into PB, while the maximal releasing rate of the gel under different pH conditions was collected as shown in Figure 3b. This shows that with the increase of the pH from 5 to 8, the releasing rate of the gel dramatically decreased from 140.28 ± 7.20 μg/h to 34.01 ± 5.62 μg/h. Meanwhile, it was found that the electrostatic interaction between tetramycin and OSA also affected the releasing behavior of the pesticide. After loading tetramycin and coumarin into the ADH-OSA gel separately, followed by soaked the gels into 20 mM pH 7.0 PB, the releasing rate of coumarin in the gel was higher than that of the tetramycin-loaded gel, this trend was also the same when the pH of the buffer was adjusted to 5.0 by HCl solution (Figure S4).

**Figure 4:**
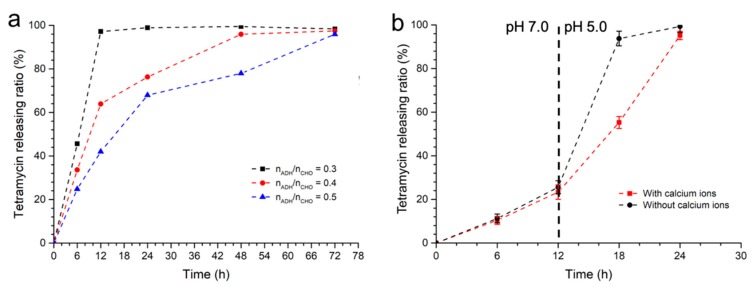
(**a**) The tetramycin releasing rate of the gel with different ADH amounts under pH 5. (**b**) The change of tetramycin releasing rate when the gel was fabricated with or without Ca^2+^. n_ADH_ and n_CHO_ are the molar quantity of ADH and the total CHO group in the gel, respectively.

At the same time, as the change of the ADH amount in the gel affected the crosslinking degree of the gel, the n_ADH_/n_CHO_ ratio of the gel also affected the releasing rate of the tetramycin. As shown in Figure 4a, when the n_ADH_/n_CHO_ was increased from 0.3 to 0.5, after being immersed in PB at pH 5, the releasing ratio of the tetramycin molecules in the gel decreased from 98% to 40% after the first 12 h. Meanwhile, the releasing rate of the tetramycin molecules in the gel with different ADH amounts also showed the same trend. Furthermore, it required 72 h for the gels to release 97% of the tetramycin when the n_ADH_/n_CHO_ was 0.4 and 0.5, respectively. This was mainly due to the fact that on the one hand, more ADH molecules in the gel would lead to more crosslinking points and a denser the gel network, which would hinder the movement of the pesticide molecules, whereas on the other hand, the increase of the amount of crosslinking points would also increase the time needed to hydrolyze all of the Schiff base structures, and thus also decrease the releasing rate of the pesticides.

The existence of Ca^2+^ in the gel may also affect the pH-sensitive pesticide releasing behavior of the gel. To study the effect of environmental pH change and Ca^2+^ on the release of pesticide, the releasing rate change of tetramycin by the gel (n_ADH_/n_CHO_ = 0.3) with or without Ca^2+^ was observed. In Figure 4b, it is shown that when the environmental pH was 7, the releasing rates of the gel with or without Ca^2+^ were similar to each other, whereas the releasing rate of the gel without Ca^2+^ was a little higher than that of the gel with Ca^2+^. When the environmental pH was changed from 7 to 5, the tetramycin in the gel without Ca^2+^ was quickly and totally released in 6 h, indicating that the change of the pH would lead to a rapid releasing of the loaded pesticide. To the gel with Ca^2+^, however, the change of pH also made the releasing rate increase; however, when the rapid releasing of the pesticide was not observed, the tetramycin was totally released in 12 h after the pH change. This result indicated that the pH change could efficiently affect the releasing rate of the gel, whereas the existence of the Ca^2+^ crosslinking point could avoid the rapid releasing of the pesticide induced by the quick hydrolysis of the Schiff base crosslinking point. This pH-dependent pesticide releasing with a stable releasing rate of ADH-OSA gel was relatively useful in the management of *R. solanacearum*. During the application, this pH-dependent pesticide releasing behavior can auto-adjust the pesticide releasing rate in the soil according to *R. solanacearum* growth. Meanwhile, a stable pesticide releasing rate could also elongate the application time of the gel, especially during the rainy season.

### 2.3. pH-Dependent Inhibition of R. solanacearum Growth of Tetramycin Loaded ADH-OSA Gel

**Figure 5:**
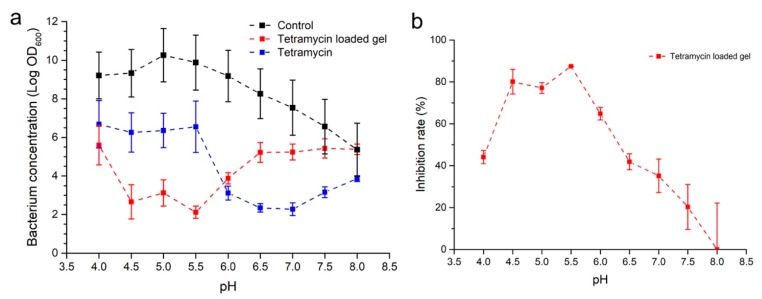
Inhibition activity of tetramycin and tetramycin-loaded ADH-OSA gel to *Ralstonia solanacearum*. (**a**) Effect on colony density of *R. solanacearum* after treating by tetramycin and tetramycin gel at different pH conditions. (**b**) The inhibition rate change of tetramycin-loaded gel under different pH conditions.

Because of the pH-dependent pesticide ability of the ADH-OSA gel, and the high releasing rate of the gel under pH 5, the gel with a n_ADH_/n_CHO_ ratio of 0.3 was chosen to study the inhibition of *R. solanacearum* under different pH conditions. Herein, pure tetramycin and tetramycin-loaded ADH-OSA gel was added separately into the medium of *R. solanacearum* with different pH values from 4.0 to 8.0 (Figure 5). It could be seen that the growth of *R. solanacearum* was closely related to the environmental pH. When the pH changed from 4.0 to 8.0, the log OD_600_ firstly increased, and the peak value then appeared when the pH was 5.0, then decreasing gradually until the pH was 8.0. Simultaneously, compared with the control group, pure tetramycin showed a moderate effect when the pH was between 4.0 and 5.5, and the inhibition rate was increased when the pH was above 6.0 (Figure 5a). On the contrary, tetramycin-loaded ADH-OSA gel showed an obvious pH-dependent inhibition activity to the bacterium growth (Figure 5b). It could be found that when the environmental pH varied from 4.0 to 8.0, the inhibition rate firstly increased to around 85% and then decreased to about 20%. Benefiting from the high pesticide releasing rate, a plateau of the inhibition rate of tetramycin-loaded gel could be observed when the pH was between 4.5 and 5.5; then, the inhibition activity of the gel decreased because of the low pesticide releasing rate of the gel under high pH conditions. Meanwhile, an interesting observation occurred when the inhibition activity of the tetramycin-loaded gel was stronger than pure tetramycin at pH 4.5, 5.0, and 5.5, which may have been due to the enrichment effect of OSA on the tetramycin molecules, induced by the electrostatic interaction [34]. When the pH was further decreased to 4.0, the colony density was again increased to the same level as that of the pure tetramycin, which may have been due to the deionization of carboxyl groups of OSA, leading to a disability in the enrichment of the tetramycin onto OSA via electrostatic interaction [35] (Figure S5). Meanwhile, compared with the colony density of the control group under different pH conditions, it could also be found that the pH range of the gel with the highest inhibition activity overlapped with the pH range of the highest colony density of *R. solanacearum*, indicating that the pesticide releasing rate of tetramycin-loaded ADH-OSA gel could respond to the *R. solanacearum* growth-induced soil pH change, and could be effectively applied in the prevention of bacterial wilt during nightshade plant production.

### 2.4. Antibacterial Activity of Tetramycin Gel, Tetramycin, and Difenoconazole

**Figure 6:**
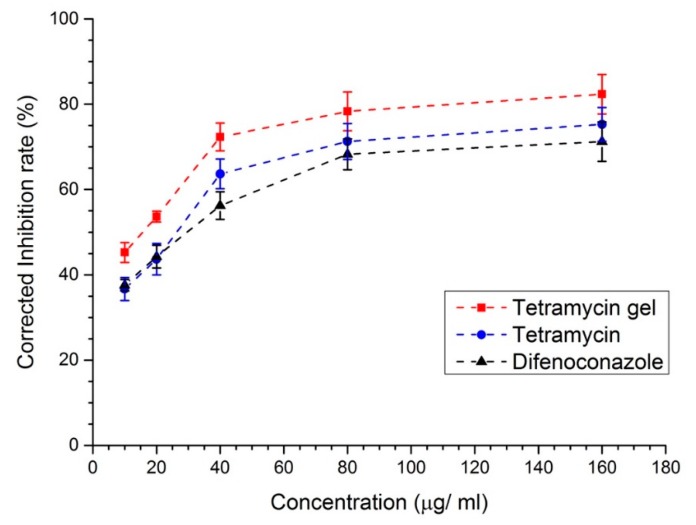
The corrected inhibition rate of *R. solanacearum* after tetramycin loaded gel, pure tetramycin, and difenoconazole was added under different concentrations at pH 5.0.

Difenoconalzole is one of the industrial pesticides that can be used in the prevention of bacterial wilt [36,37]. Here, the anti-bacterial activities of tetramycin-loaded ADH-OSA gel, pure tetramycin, and difenoconazole under different total pesticide concentrations from 10 μg/mL to 160 μg/mL were compared (Figure 6 and Figure S6). The results showed that the anti-bacterial activities of both pure tetramycin and tetramycin-loaded ADH-OSA gel were higher than difenoconazole under every concentration when the environmental pH was 5. Also, tetramycin-loaded ADH-OSA gel was more effective than pure tetramycin, which might be the enrichment effect leaded by the gel to the tetramycin molecules. Furthermore, it was noteworthy that when tetramycin concentration was 160 μg/mL, the corrected inhibition rate of tetramycin-loaded ADH-OSA gel could reach as high as 82%, higher than that of pure tetramycin (73%) and difenoconazole (68%). This result proved that tetramycin-loaded ADH-OSA gel had a potential ability to manage bacterial wilt.

## 3. Methods and Experiments

### 3.1. Materials

Sodium alginate (98%), HCl, glycol, NaCl (99%), sodium periodate (98%), and NaOH (99%) were purchased form Adamas (Minneapolis, MN, USA). The double distillated H_2_O (ddH_2_O) was purified by a Synergy (Milipore, USA). The gel was characterized by a Nicolet iS 10 Fourier transform inferred spectrometer (ThermoFisher, Waltham, MA, USA). The *R. solanacearum* (phylotype I, race 1, biovar 3) tobacco strain was used in this study [38]. The bacterial pathogen was grown at 30 °C in rich B medium [39]. Tetramycin was presented by Liaoning Wkioc Bioengineering Co., Ltd., Benxi, China. Difenoconazole used as positive control was purchased from Jiangsu Sevencontinent Green Chemical Co., Ltd., Zhangjiagang, China [38,39].

### 3.2. Oxidation of Sodium Alginate

The OSA was prepared from sodium alginate according to the literature [33]. Sodium alginate (SA, 5 g) was added into 100 mL ethanol and stirred for 5 min until being dissolved. Then, 1 mL 8 vt% HCl was added into the solvent. Sodium periodate (5 g) was added into 100 mL ddH_2_O and the solution was added into the SA mixture to oxidize SA. The reaction was stirred for 18 h under room temperature, then 5 mL glycol and 1 g NaCl was added into the mixture to stop the reaction. The mixture was then dialyzed for 3 days and freeze dried to get the pure product. The oxidation degree of the produced OSA was calculated by conductance titration and was controlled at 0.48.

### 3.3. Preparation of Tetramycin Loaded ADH-OSA Gel

ADH-OSA gel was prepared as follows: OSA was added into PB (200 mM, pH 7.5) until the concentration reached 25 mg/mL. Then, ADH solutions with different concentration (70 mM, 93 mM, and 117 mM) was added into the OSA solution quickly and stirred for 15 s; the volume ratio of OSA solution and ADH solution was 13:7. The mixture was then poured into the mold and held for 30 min to form the pre-gel. The pre-gel was then immersed into 0.2 M CaCl_2_ for 5 min, and washed by PB (200 mM, pH 7.5), and the gel was placed under room temperature for 6 h to produce the ADH-OSA gel. The formation of hydrazine bonds in the gel was monitored by an FTIR spectrometer.

The standard curve of tetramycin was firstly prepared before the fabrication of the tetramycin-loaded ADH-OSA gel. To make the standard curve of tetramycin, tetramycin solution with different tetramycin concentrations (0 mg/mL, 25 mg/mL, 50 mg/mL, 200 mg/mL, and 400 mg/mL) were prepared, and the UV-VIS absorbance data between the wavelength of 200 nm to 350 nm of the solution was collected by a UV-VIS spectrometer. Then, the absorbance at the wavelength of 272 nm of tetramycin with different concentration was linearly fitted. The absorption coefficient was calculated according to the slope of the curve, and the concentration of the tetramycin could be calculated via the Lambert–Beer method.

The tetramycin-loaded ADH-OSA gel was prepared in the same way as the ADH-OSA gel, but before the mixing of the ADH solution and OSA solution, the tetramycin solution with different tetramycin concentration was added into the OSA solution; the volume ratio of OSA, ADH, and tetramycin solution was 13:7:4. The loading content and loading amount of tetramycin in the ADH-OSA gel was calculated by Equations (1) and (2):

(1) $$LC=\frac{c_{Tetramycin}\;\times \;v_{Tetramycin}}{w_{\mathrm{ADH-OSA}\;gel}}\;\times \;100\%,$$

(2) $$LA=\frac{c_{Tetramycin}\;\times \;v_{Tetramycin}}{v_{\mathrm{ADH-OSA}\;gel}}$$

where the *c_Tetramycin_*, *v_Tetramycin_*, *w_ADH-OSA gel_*, and *v_ADH-OSA gel_* are, respectively, the concentration and volume of the tetramycin solution added into the ADH-OSA gel, and the final weight and volume of tetramycin loaded ADH-OSA gel.

### 3.4. Calculation of Tetramycin Releasing Rate

To calculate the tetramycin releasing rate, 2 mL tetramycin-loaded ADH-OSA gel was prepared, containing 2 mg tetramycin inside. Then, the gel was immersed into the 150 mL PB with different pH values (3, 4, 5, 6, and 7). Then, 0.5 mL soaking solution of the gel was taken from the mixture and diluted to calculate the tetramycin concentration via its light absorbance at the wavelength of 272 nm according to the standard curve of the tetramycin after a certain period.

### 3.5. Assessment of Colony Density of R. solanacearum

To evaluate disease development under different pH conditions (4.0–8.0), we performed an indoor pot experiment. The pH of B medium was adjusted using different concentrations of hydrochloric acid or NaOH.

The growth curve of *R. solanacearum* under different pH conditions (4.0∼8.0) was determined as follows: First, *R. solanacearum* cells were grown in B medium at 28 °C for 12 h. The bacterial suspension (log OD_600_ ≈ 1.0) was then transferred into 100 mL pH-adjusted B medium at a 1:100 ratio, and 2 mg tetramycin, DMSO, and 2 mL tetramycin-loaded ADH-OSA gel (1 mg/mL) were separately added into the medium. The OD_600_ of each treatment was measured using a spectrophotometer with a 4 h time interval. The bacterial concentration was determined using a nephelometer. Three independent experiments were performed, and three replicates were used for each treatment in each independent experiment. The corrected inhibition rate could be calculated by the following formula:

(3) $$P=\frac{A_{0}-A_{1}}{A_{0}}\times 100\%$$

where *P* is the corrected inhibition rate, *A*_0_ is the increased log OD_600_ value of the control treatment, and *A*_1_ is the increased log OD_600_ value of pesticide treatments.

### 3.6. Antibacterial Activity of Tetramycin Gel and Difenoconazole

The antibacterial activity of tetramycin, tetramycin gel, and difenoconazole against *R. solanacearum* were individually tested in vitro through a filter paper disc agar diffusion method with minor modifications [40]. A suspension (100 µL) containing 108 CFU/mL *R. solanacearum* was inoculated directly onto each NA medium. The pH of the NA medium was adjusted to 5 using different concentrations of hydrochloric acid or NaOH. They were also watered with pH-adjusted water to create fixed pH conditions. An Oxford cup (Φ6 × 10 mm) was applied to the surface of the agar plates. The stock solutions of tetramycin and difenoconazole, as well as the soaking solution of the tetramycin loaded ADH-OSA gel, were diluted to 160, 80, 40, 20, 10 μg/mL, independently. Then, 10 µL dilutions were dropped on the plate. DMSO (16 mg/mL) was used as control. The plates were incubated at 30 ± 1 °C for 24 h. The diameters of the inhibition zones, except for the 6 mm disc diameter, were measured. Experiments were performed in triplicate, and the results were reported as mean values. The turbidity of each treatment was measured before culture. When the logarithmic growth period of the control treatment was reached, the turbidity of each treatment was measured and recorded. The corrected inhibition rate could be calculated by the following formula:

(4) $$P=\frac{T_{0}-T_{1}}{T_{0}}\times 100\%$$

where *P* is the corrected inhibition rate, *A*_0_ is the increased turbidity of the control treatment, and *A*_1_ is the increased turbidity of pesticide treatments.

## 4. Conclusions

In conclusion, a new double-crosslinked ADH-OSA gel with pH-dependent pesticide releasing behavior is reported in this study, being based on the pH-sensitive Schiff base reaction between OSA and ADH and the crosslinking of Ca^2+^ to the OSA. The Schiff base crosslinking point can provide a gel pH-dependent pesticide releasing ability, whereas the Ca^2+^ crosslinking point can avoid the rapid releasing of the pesticide during environmental pH change. Further study showed that the gel could obviously increase its pesticide releasing rate when the environmental pH was decreased from 7 to 5. Such pH-dependent pesticide releasing behavior coincided with the soil pH change during the bacterial wilt of nightshade production. The tetramycin was loaded into ADH-OSA gel and performed a pH-dependent inhibition to the growth of *R. solanacearum*. It was also proven that this tetramycin-loaded ADH-OSA gel was more efficient than both pure tetramycin and difenoconazole when used to inhibit the growth of *R. solanacearum*—the maximum inhibition rate of the gel could even reach as high as 82%. Thus, considering the auto-adapted pesticide releasing ability of the tetramycin-loaded ADH-OSA gel and its high anti-bacterial activity against *R. solanacearum*, it is believed that this pesticide gel can have a high application potential in the controlling of bacterial wilt. Meanwhile, this gel can also potentially provide a smart pesticide carrier in the management of plant disease for accurate pesticide application during agriculture production.

## Supplementary Materials

The following are available online at https://www.mdpi.com/1420-3049/24/19/3606/s1, Figure S1: The storage modulus (G’), loss modulus (G’’) and Tan δ of the gel with (a) n_ADH_/n_CHO_ = 0.3, (b) n_ADH_/n_CHO_ = 0.4 and (c) n_ADH_/n_CHO_ = 0.5. Figure S2: The change of leachate pH after merging ADH-OSA gel into ddH_2_O. Figure S3: The standard curve of Tetramycin in ddH_2_O. Figure S4: The releasing of tetramycin and coumarin loaded in the ADH-OSA gel, the pH of the solution was changed from 7 to 5 after merging the gel for 12 h. Figure S5: Images of *R. solanacearum* growth with tetramycin loaded gel and pure tetramycin respectively under different environmental pH. Figure S6: Images of Petri dish of *R. solanacearum* after being added tetramycin loaded gel, pure tetramycin and difenoconazole under different concentrations at pH 5.0.
