# Supplementary material for: Fabrication of pH-Sensitive Tetramycin Releasing Gel and Its Antibacterial Bioactivity against Ralstonia solanacearum
Source: Molecules. 2019 Oct 7;24(19):3606. doi: 10.3390/molecules24193606 (PMC6804146; doi:10.3390/molecules24193606)
Supplement: Supplementary file 1 [file molecules-24-03606-s001.zip › molecules-591145-SI.pdf]

Supporting Information

Fabrication of pH-Sensitive Tetramycin Releasing Gel and Its Antibacterial Bioactivity against *Ralstonia solanacearum*

Xiaozhou Ma [1,2,†], Shunyu Xiang [2,3,†], Huijun Xie [3], Linhai He [1], Xianchao Sun [2,3], Yongqiang Zhang [2,3,*] and Jin Huang [1,2,*]

[1] School of Chemistry and Chemical Engineering, Southwest University, Chongqing 400715, China; maxiaozhou@swu.edu.cn (X.M.); h996130557@163.com (L.H.)

[2] Chongqing Key Laboratory of Soft-Matter Material Chemistry and Function Manufacturing, Southwest University, Chongqing 400715 China; xiangshunyu0325@163.com (S.X.); sunxianchao@163.com (X.S.)

[3] College of Plant Protection, State Cultivation Base of Crop Stress Biology for Southern Mountainous Land, Southwest University, Chongqing 400715 China; xiehj941019@163.com (H.X)

† The authors contributed equally to this work.

* Correspondence: zyqiang@swu.edu.cn (Y.Z.); huangjin2015@swu.edu.cn (J.H.)

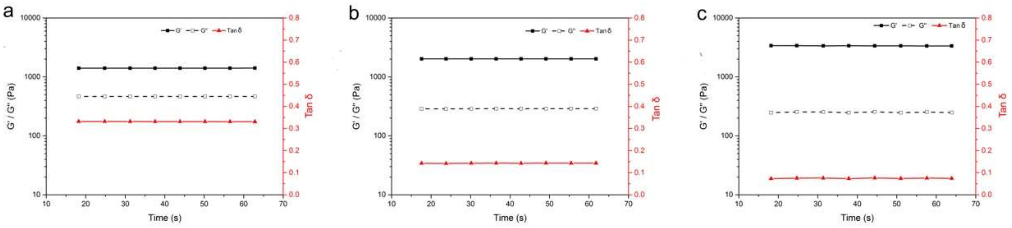

Figure S1. The storage modulus (G′), loss modulus (G′′) and Tan δ of the gel with (a) $n_{ADH}/n_{CHO} = 0.3$, (b) $n_{ADH}/n_{CHO} = 0.4$ and (c) $n_{ADH}/n_{CHO} = 0.5$.

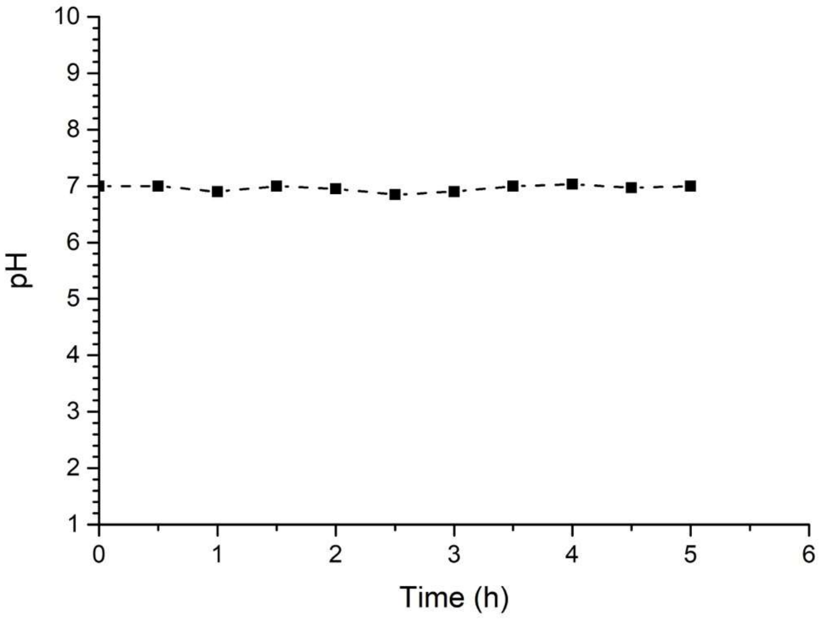

Figure S2. The change of leachate pH after merging ADH-OSA gel into ddH_2O.

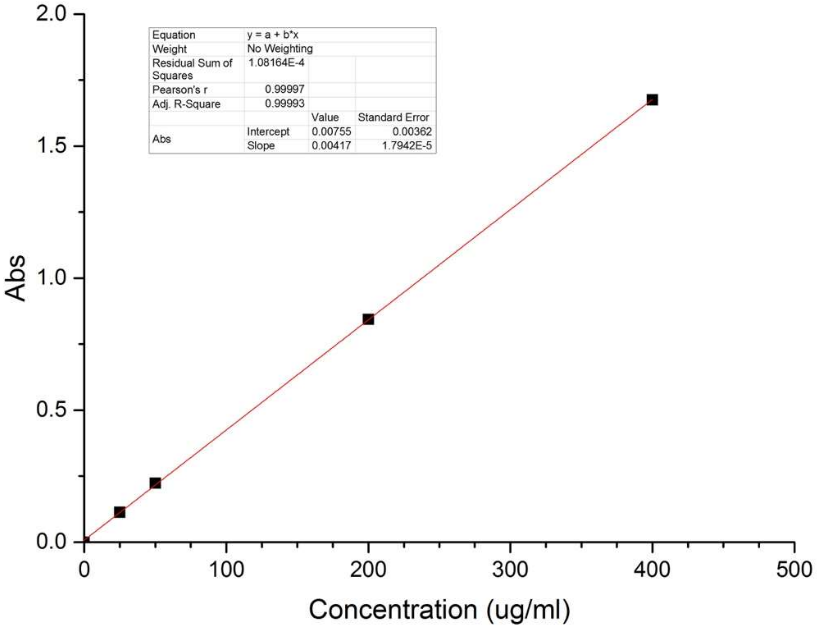

Figure S3. The standard curve of Tetramycin in ddH_2O.

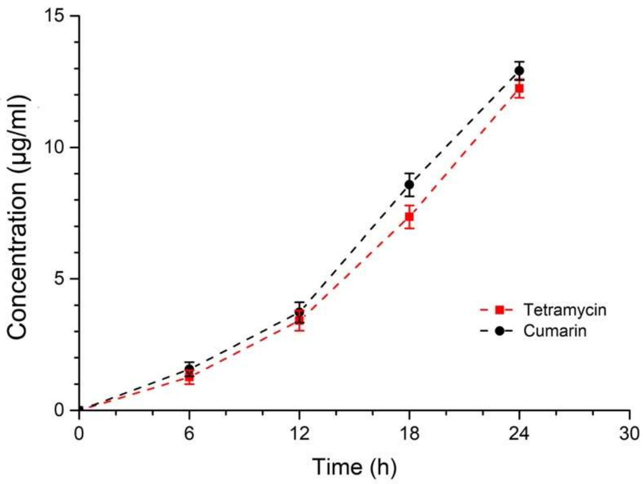

Figure S4. The releasing of tetramycin and coumarin loaded in the ADH-OSA gel, the pH of the solution was changed from 7 to 5 after merging the gel for 12 h.

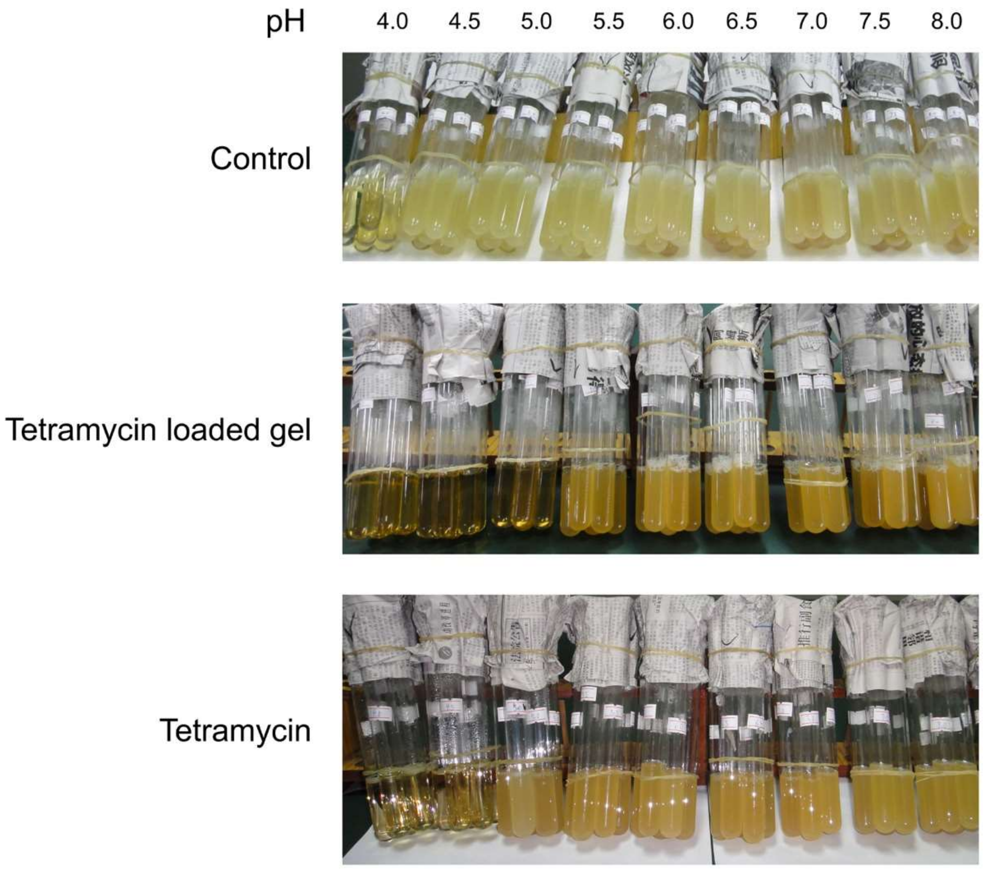

Figure S5. Images of *R. solanacearum* growth with tetramycin loaded gel and pure tetramycin respectively under different environmental pH.

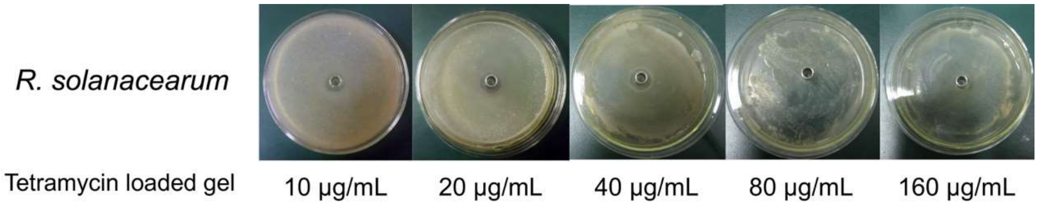

Figure S6. Images of Petri dish of *R. solanacearum* after being added tetramycin loaded gel, pure tetramycin and difenoconazole under different concentrations at pH 5.0.
